# 2-(1,3-Dioxoisoindolin-2-yl)acetic acid–*N*′-[(*E*)-2-meth­oxy­benzyl­idene]pyridine-4-carbohydrazide (1/1)

**DOI:** 10.1107/S1600536812031388

**Published:** 2012-07-14

**Authors:** Shaaban K. Mohamed, Muhammad Akhyar Farrukh, Mehmet Akkurt, Mustafa R. Albayati, Antar A. Abdelhamid

**Affiliations:** a Chemistry and Environmental Division, Manchester Metropolitan University, Manchester M1 5GD, England; b Department of Chemistry, Government College University, Lahore 54000, Pakistan; c Department of Physics, Faculty of Sciences, Erciyes University, 38039 Kayseri, Turkey; d Chemistry Department, Tikrit University, Tikrit, Iraq

## Abstract

In the title 1:1 cocrystal, C_10_H_7_NO_4_·C_14_H_13_N_3_O_2_, mol­ecules are linked by inter­molecular C—H⋯O, N—H⋯O and O—H⋯N hydrogen bonds, forming a three-dimensional network. In addition, π–π stacking inter­actions [with centroid–centroid distances of 3.5723 (19) and 3.6158 (18) Å] are observed.

## Related literature

For the use of co-crystals in drug design and delivery, see: Vishweshwar *et al.* (2009 ▶); Peterson *et al.* (2006 ▶); McNamara *et al.* (2006 ▶). For anti-tuberculosis drugs containing the isoniazid core structure, see: Bijev (2006 ▶); Imramovský *et al.* (2007 ▶); Maccari *et al.* (2005 ▶); Schultheiss & Newman (2009 ▶); Shindikar & Viswanathan (2005 ▶); Sinha *et al.* (2005 ▶); Sriram *et al.* (2006 ▶).
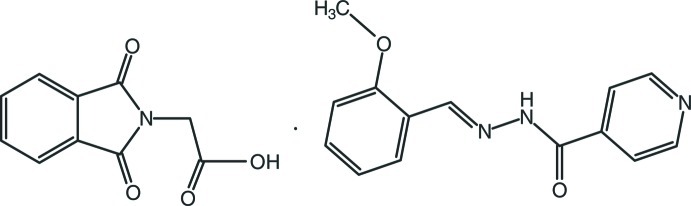


## Experimental

### 

#### Crystal data

C_10_H_7_NO_4_·C_14_H_13_N_3_O_2_
*M* *_r_* = 460.44
Monoclinic, 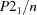

*a* = 7.0747 (10) Å
*b* = 43.511 (6) Å
*c* = 7.5477 (9) Å
β = 110.015 (5)°
*V* = 2183.1 (5) Å^3^
*Z* = 4
Mo *K*α radiation
μ = 0.10 mm^−1^
*T* = 296 K
0.30 × 0.20 × 0.20 mm

#### Data collection

Bruker Kappa APEXII CCD diffractometer
Absorption correction: multi-scan (*SADABS*; Bruker, 2005 ▶) *T*_min_ = 0.970, *T*_max_ = 0.980
19892 measured reflections
4304 independent reflections
2345 reflections with *I* > 2σ(*I*)
*R*_int_ = 0.061

#### Refinement

*R*[*F* ^2^ > 2σ(*F* ^2^)] = 0.055
*wR*(*F* ^2^) = 0.135
*S* = 1.02
4304 reflections
309 parameters
H-atom parameters constrained
Δρ_max_ = 0.14 e Å^−3^
Δρ_min_ = −0.18 e Å^−3^

### 

Data collection: *APEX2* (Bruker, 2009 ▶); cell refinement: *SAINT* (Bruker, 2009 ▶); data reduction: *SAINT*; program(s) used to solve structure: *SHELXS97* (Sheldrick, 2008 ▶); program(s) used to refine structure: *SHELXL97* (Sheldrick, 2008 ▶); molecular graphics: *ORTEP-3 for Windows* (Farrugia, 1997 ▶); software used to prepare material for publication: *WinGX* (Farrugia, 1999 ▶) and *PLATON* (Spek, 2009 ▶).

## Supplementary Material

Crystal structure: contains datablock(s) global, I. DOI: 10.1107/S1600536812031388/qm2075sup1.cif

Structure factors: contains datablock(s) I. DOI: 10.1107/S1600536812031388/qm2075Isup2.hkl

Supplementary material file. DOI: 10.1107/S1600536812031388/qm2075Isup3.cml

Additional supplementary materials:  crystallographic information; 3D view; checkCIF report

## Figures and Tables

**Table 1:** Hydrogen-bond geometry (Å, °)

*D*—H⋯*A*	*D*—H	H⋯*A*	*D*⋯*A*	*D*—H⋯*A*
O3—H3*A*⋯N4^i^	0.82	1.86	2.681 (3)	177
N3—H3*B*⋯O6^ii^	0.86	2.12	2.958 (3)	164
C4—H4⋯O4^iii^	0.93	2.44	3.190 (4)	138
C20—H20⋯O3^ii^	0.93	2.57	3.500 (4)	174

Symmetry codes: (i) 
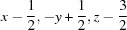
; (ii) 
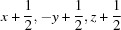
; (iii) 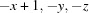.

**Fig. 1.**
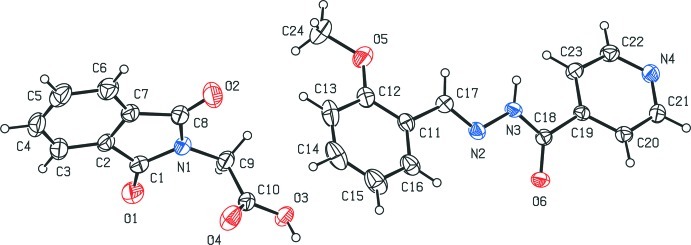
The molecular structure of (I) with the atom numbering scheme. Displacement ellipsoids for non-H atoms are drawn at the 30% probability level.

**Fig. 2.**
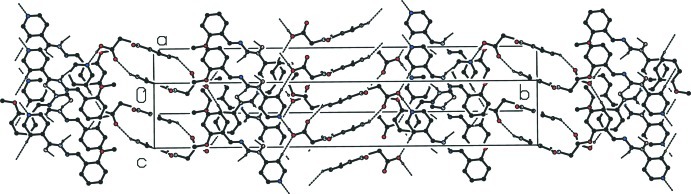
The molecular packing and the hydrogen bonding viewed along the c axis. Hydrogen atoms not involved in hydrogen bonding have been omitted for clarity.

## supplementary crystallographic information

### Comment

The use of co-crystals in drug design and delivery and as functional materials with potential applications as pharmaceuticals has recently attracted considerable interest (Vishweshwar *et al.*, 2009; Peterson *et al.*, 2006; McNamara *et al.*, 2006). Moreover, co-crystallization in particular is a reliable method for the modification of drug physical and technical properties such as solubility, dissolution rate, stability, hygroscopisity and compressibility without alternating the pharmacological behaviour of their ingredients (Schultheiss & Newman, 2009). Compounds incorporating the isoniazid (INH) core structure have shown high inhibitory activity *in vitro* (Bijev, 2006; Imramovský *et al.*, 2007) and in mice towards *M. tuberculosis* H37Rv, ATCC 27294, *M. tuberculosis* clinical isolates and isoniazid -resistant *M. tuberculosis* (Maccari *et al.*, 2005; Shindikar & Viswanathan, 2005; Sinha *et al.*, 2005; Sriram *et al.*, 2006). In this context and on continuation of our interest in the synthesis of potentially biologically active molecules based on the core structure of isoniazid we decided to investigate the reaction of isoniazid-related hydrazones with phthalimido-acetic acid. The reaction showed the unexpected co-crystallized product (I) with its ingridients in a 1:1 ratio. In this study we report a new co-crystallization method for the anti-tubercular drug *N*'-[(*E*)-(4-methoxyphenyl)methylidene]pyridine-4-carbohydrazide with (1,3-dioxo-1,3-dihydro-2*H*-isoindol-2-yl)acetic acid and the crystal structure of their cocrystal compound.

Fig. 1 shows the molecules of a 1:1 *cocrystal* of (1,3-dioxo-1,3-dihydro-2*H*-isoindol-2-yl)acetic acid and *N*'-[(1E)-(2-methoxyphenyl)methylidene]pyridine-4-carbohydrazide. The bond lengths and bond angles are all within the expected ranges. In the molecule of (1,3-dioxo-1,3-dihydro-2*H*-isoindol-2-yl)acetic acid, the 2,3-dihydro-1*H*-isoindole ring (N1/C1–C8) is planar with a maimum deviation of 0.014 (3) Å for C2 atom. In the molecule of *N*'-[(1E)-(2-methoxyphenyl)methylidene]pyridine-4-carbohydrazide, the C11–C16 benzene and N4C19–C23 pyridine rings make a dihedral angle of 4.44 (15)° with each other.

The crystal structure is stabilized by intermolecular C—H···O, N—H···O and O—H···N hydrogen bonds, forming a three dimensional network (Table 1, Fig. 2). Furthermore, π-π stacking interactions [*Cg*3···*Cg*4(1 - *x*, -*y*, 1 - *z*) = 3.5723 (19) Å and *Cg*4···*Cg*4(1 - *x*, -*y*, -*z*) = 3.6158 (18) Å; where *Cg*3 and *Cg*4 are centroids of the N1/C1/C2/C7/C8 and C1–C6 rings, respectively] contribute to stabilize the crystal structure.

### Experimental

A mixture of 255 mg (1 mmol) *N*'-[(*E*)-(2-methoxyphenyl)methylidene]pyridine-4-carbohydrazide and 205 mg (1 mmol) (1,3-dioxo-1,3-dihydro-2*H*-isoindol-2-yl)acetic acid in 50 ml e thanol was refluxed at 351 K for six hours. The reaction mixture was poured onto crushed ice to afford a solid product which was filtered off, washed with ethanol dried under vacuum and recrystallized from ethanol in good yield (78%). Crystals (m.p. 449 K) suitable for X-ray diffraction were grown by slow evaporation from an ethanol solution at room temperature over 24 h.

### Refinement

H-atoms were placed in calculated positions [O—H = 0.82 Å, N—H = 0.86 Å, C–H = 0.93–0.97 Å] and were included in the refinement in the riding model approximation, with *U_iso_*(H) = 1.2 or 1.5*U_eq_*(C, N, O).

### Crystal data

**Table:**

C_10_H_7_NO_4_·C_14_H_13_N_3_O_2_	*F*(000) = 960
*M**_r_* = 460.44	*D*_x_ = 1.401 Mg m^−^^3^
Monoclinic, *P*2_1_/*n*	Mo *K*α radiation, λ = 0.71073 Å
Hall symbol: -P 2yn	Cell parameters from 410 reflections
*a* = 7.0747 (10) Å	θ = 2.8–18.3°
*b* = 43.511 (6) Å	µ = 0.10 mm^−^^1^
*c* = 7.5477 (9) Å	*T* = 296 K
β = 110.015 (5)°	Prism, white
*V* = 2183.1 (5) Å^3^	0.30 × 0.20 × 0.20 mm
*Z* = 4	

### Data collection

**Table:**

Bruker Kappa APEXII CCD diffractometer	4304 independent reflections
Radiation source: fine-focus sealed tube	2345 reflections with *I* > 2σ(*I*)
Graphite monochromator	*R*_int_ = 0.061
ω scans	θ_max_ = 26.0°, θ_min_ = 1.9°
Absorption correction: multi-scan (*SADABS*; Bruker, 2005)	*h* = −8→8
*T*_min_ = 0.970, *T*_max_ = 0.980	*k* = −53→53
19892 measured reflections	*l* = −9→6

### Refinement

**Table:**

Refinement on *F*^2^	Primary atom site location: structure-invariant direct methods
Least-squares matrix: full	Secondary atom site location: difference Fourier map
*R*[*F*^2^ > 2σ(*F*^2^)] = 0.055	Hydrogen site location: inferred from neighbouring sites
*wR*(*F*^2^) = 0.135	H-atom parameters constrained
*S* = 1.02	*w* = 1/[σ^2^(*F*_o_^2^) + (0.0485*P*)^2^ + 0.2903*P*] where *P* = (*F*_o_^2^ + 2*F*_c_^2^)/3
4304 reflections	(Δ/σ)_max_ < 0.001
309 parameters	Δρ_max_ = 0.14 e Å^−^^3^
0 restraints	Δρ_min_ = −0.18 e Å^−^^3^

### Special details

**Table:**

Geometry. Bond distances, angles *etc*. have been calculated using the rounded fractional coordinates. All su's are estimated from the variances of the (full) variance-covariance matrix. The cell e.s.d.'s are taken into account in the estimation of distances, angles and torsion angles
Refinement. Refinement on *F*^2^ for ALL reflections except those flagged by the user for potential systematic errors. Weighted *R*-factors *wR* and all goodnesses of fit *S* are based on *F*^2^, conventional *R*-factors *R* are based on *F*, with *F* set to zero for negative *F*^2^. The observed criterion of *F*^2^ > σ(*F*^2^) is used only for calculating -*R*-factor-obs *etc*. and is not relevant to the choice of reflections for refinement. *R*-factors based on *F*^2^ are statistically about twice as large as those based on *F*, and *R*-factors based on ALL data will be even larger.

### Fractional atomic coordinates and isotropic or equivalent isotropic displacement parameters (Å^2^)

**Table:**

	*x*	*y*	*z*	*U*_iso_*/*U*_eq_	
O5	0.9659 (3)	0.13677 (4)	0.9365 (3)	0.0713 (9)	
O6	0.7705 (3)	0.28342 (4)	0.8630 (2)	0.0491 (7)	
N2	0.9227 (3)	0.22710 (5)	0.8728 (3)	0.0448 (8)	
N3	0.9636 (3)	0.24511 (5)	1.0332 (3)	0.0431 (8)	
N4	0.9687 (3)	0.32666 (5)	1.5100 (3)	0.0475 (9)	
C11	0.9448 (4)	0.17874 (6)	0.7399 (4)	0.0426 (10)	
C12	0.9519 (4)	0.14724 (6)	0.7626 (4)	0.0527 (11)	
C13	0.9412 (5)	0.12788 (7)	0.6119 (5)	0.0733 (14)	
C14	0.9201 (5)	0.14100 (10)	0.4399 (5)	0.0838 (16)	
C15	0.9080 (5)	0.17224 (9)	0.4141 (5)	0.0780 (16)	
C16	0.9193 (4)	0.19068 (7)	0.5638 (4)	0.0574 (11)	
C17	0.9700 (4)	0.19899 (6)	0.9011 (4)	0.0425 (9)	
C18	0.8817 (4)	0.27317 (6)	1.0142 (4)	0.0383 (9)	
C19	0.9250 (3)	0.29134 (5)	1.1918 (3)	0.0345 (8)	
C20	0.9321 (4)	0.32285 (6)	1.1838 (4)	0.0425 (9)	
C21	0.9562 (4)	0.33942 (6)	1.3457 (4)	0.0478 (10)	
C22	0.9617 (4)	0.29627 (6)	1.5154 (4)	0.0474 (10)	
C23	0.9432 (4)	0.27773 (6)	1.3624 (3)	0.0432 (9)	
C24	0.9578 (6)	0.10465 (7)	0.9630 (5)	0.0989 (18)	
O1	0.1383 (4)	0.04421 (5)	0.1887 (3)	0.0801 (9)	
O2	0.8106 (4)	0.05255 (5)	0.4959 (3)	0.0820 (9)	
O3	0.4269 (3)	0.13691 (4)	0.2806 (3)	0.0563 (7)	
O4	0.5019 (4)	0.09977 (4)	0.1147 (3)	0.0768 (9)	
N1	0.4681 (4)	0.05545 (5)	0.3577 (3)	0.0530 (9)	
C1	0.3116 (5)	0.03656 (7)	0.2495 (4)	0.0554 (11)	
C2	0.4075 (5)	0.00722 (6)	0.2291 (4)	0.0524 (10)	
C3	0.3233 (5)	−0.01899 (7)	0.1349 (4)	0.0665 (14)	
C4	0.4514 (7)	−0.04291 (7)	0.1340 (4)	0.0748 (14)	
C5	0.6539 (7)	−0.04044 (7)	0.2267 (5)	0.0771 (14)	
C6	0.7384 (6)	−0.01406 (7)	0.3214 (4)	0.0711 (14)	
C7	0.6119 (5)	0.00973 (6)	0.3213 (4)	0.0528 (10)	
C8	0.6528 (5)	0.04091 (7)	0.4045 (4)	0.0562 (11)	
C9	0.4417 (5)	0.08717 (6)	0.3977 (4)	0.0571 (10)	
C10	0.4610 (4)	0.10833 (6)	0.2468 (4)	0.0495 (10)	
H3B	1.03930	0.23840	1.14160	0.0520*	
H13	0.94820	0.10670	0.62710	0.0880*	
H14	0.91390	0.12830	0.33880	0.1000*	
H15	0.89240	0.18070	0.29680	0.0940*	
H16	0.90950	0.21190	0.54670	0.0690*	
H17	1.02040	0.19130	1.02330	0.0510*	
H20	0.92090	0.33280	1.07150	0.0510*	
H21	0.96430	0.36070	1.34000	0.0570*	
H22	0.96960	0.28690	1.62860	0.0570*	
H23	0.94310	0.25640	1.37370	0.0520*	
H24A	0.83290	0.09670	0.87840	0.1490*	
H24B	0.96730	0.10060	1.09080	0.1490*	
H24C	1.06770	0.09490	0.93790	0.1490*	
H3	0.18520	−0.02070	0.07370	0.0800*	
H3A	0.44340	0.14810	0.19960	0.0840*	
H4	0.39910	−0.06090	0.06940	0.0900*	
H5	0.73670	−0.05700	0.22570	0.0920*	
H6	0.87640	−0.01250	0.38320	0.0850*	
H9A	0.54170	0.09270	0.51780	0.0680*	
H9B	0.30990	0.08980	0.40850	0.0680*	

### Atomic displacement parameters (Å^2^)

**Table:**

	*U*^11^	*U*^22^	*U*^33^	*U*^12^	*U*^13^	*U*^23^
O5	0.0945 (17)	0.0458 (13)	0.0698 (15)	0.0059 (11)	0.0233 (12)	0.0036 (11)
O6	0.0610 (13)	0.0456 (11)	0.0326 (10)	0.0085 (9)	0.0054 (9)	0.0006 (8)
N2	0.0474 (14)	0.0426 (14)	0.0398 (13)	0.0021 (11)	0.0089 (10)	−0.0081 (10)
N3	0.0519 (14)	0.0403 (13)	0.0301 (12)	0.0075 (11)	0.0050 (10)	−0.0024 (10)
N4	0.0560 (16)	0.0456 (15)	0.0419 (14)	−0.0016 (11)	0.0179 (11)	−0.0047 (11)
C11	0.0352 (16)	0.0470 (17)	0.0452 (17)	−0.0030 (12)	0.0132 (12)	−0.0063 (13)
C12	0.0480 (19)	0.0502 (19)	0.0577 (19)	−0.0030 (14)	0.0154 (15)	−0.0086 (15)
C13	0.064 (2)	0.061 (2)	0.092 (3)	−0.0124 (17)	0.0228 (19)	−0.034 (2)
C14	0.079 (3)	0.108 (3)	0.068 (2)	−0.026 (2)	0.030 (2)	−0.044 (2)
C15	0.079 (3)	0.107 (3)	0.053 (2)	−0.030 (2)	0.0289 (18)	−0.019 (2)
C16	0.059 (2)	0.071 (2)	0.0436 (18)	−0.0141 (15)	0.0193 (15)	−0.0080 (15)
C17	0.0410 (16)	0.0442 (17)	0.0432 (15)	0.0010 (13)	0.0157 (12)	−0.0013 (13)
C18	0.0391 (16)	0.0390 (16)	0.0369 (15)	−0.0013 (12)	0.0131 (12)	0.0007 (12)
C19	0.0288 (14)	0.0390 (15)	0.0340 (14)	0.0002 (11)	0.0084 (11)	0.0010 (11)
C20	0.0460 (17)	0.0449 (17)	0.0360 (15)	−0.0030 (13)	0.0131 (12)	0.0019 (12)
C21	0.0545 (19)	0.0391 (16)	0.0489 (18)	−0.0064 (13)	0.0166 (14)	−0.0018 (14)
C22	0.0557 (19)	0.0493 (18)	0.0380 (15)	0.0031 (14)	0.0170 (13)	0.0041 (13)
C23	0.0527 (18)	0.0374 (15)	0.0407 (16)	0.0046 (13)	0.0177 (13)	0.0039 (12)
C24	0.125 (4)	0.047 (2)	0.114 (3)	0.001 (2)	0.027 (3)	0.014 (2)
O1	0.0718 (17)	0.0643 (15)	0.0895 (16)	0.0049 (13)	0.0088 (13)	0.0052 (12)
O2	0.0728 (17)	0.0660 (15)	0.0948 (17)	−0.0089 (13)	0.0128 (14)	−0.0199 (13)
O3	0.0881 (15)	0.0373 (11)	0.0495 (12)	0.0022 (10)	0.0312 (11)	0.0029 (9)
O4	0.136 (2)	0.0485 (13)	0.0652 (14)	0.0040 (12)	0.0592 (15)	−0.0054 (11)
N1	0.0680 (18)	0.0366 (13)	0.0507 (14)	−0.0013 (12)	0.0155 (13)	0.0014 (11)
C1	0.069 (2)	0.0473 (18)	0.0458 (17)	−0.0029 (17)	0.0143 (16)	0.0084 (14)
C2	0.080 (2)	0.0372 (17)	0.0402 (16)	−0.0030 (15)	0.0207 (16)	0.0047 (13)
C3	0.094 (3)	0.050 (2)	0.0545 (19)	−0.0154 (19)	0.0243 (18)	−0.0047 (15)
C4	0.127 (3)	0.043 (2)	0.062 (2)	−0.014 (2)	0.042 (2)	−0.0098 (16)
C5	0.119 (3)	0.048 (2)	0.075 (2)	0.013 (2)	0.047 (2)	0.0021 (18)
C6	0.087 (3)	0.055 (2)	0.071 (2)	0.0114 (19)	0.0266 (19)	−0.0008 (17)
C7	0.072 (2)	0.0431 (18)	0.0423 (16)	−0.0014 (15)	0.0181 (16)	0.0025 (13)
C8	0.071 (2)	0.0447 (18)	0.0505 (18)	−0.0034 (17)	0.0178 (17)	−0.0006 (14)
C9	0.083 (2)	0.0366 (16)	0.0546 (18)	−0.0009 (15)	0.0275 (16)	0.0006 (13)
C10	0.063 (2)	0.0380 (17)	0.0460 (17)	−0.0006 (13)	0.0168 (15)	−0.0031 (14)

### Geometric parameters (Å, º)

**Table:**

O5—C12	1.361 (4)	C22—C23	1.378 (4)
O5—C24	1.416 (4)	C13—H13	0.9300
O6—C18	1.227 (3)	C14—H14	0.9300
O1—C1	1.200 (5)	C15—H15	0.9300
O2—C8	1.205 (4)	C16—H16	0.9300
O3—C10	1.308 (3)	C17—H17	0.9300
O4—C10	1.189 (4)	C20—H20	0.9300
O3—H3A	0.8200	C21—H21	0.9300
N2—N3	1.387 (3)	C22—H22	0.9300
N2—C17	1.267 (3)	C23—H23	0.9300
N3—C18	1.338 (3)	C24—H24A	0.9600
N4—C21	1.334 (4)	C24—H24B	0.9600
N4—C22	1.324 (3)	C24—H24C	0.9600
N3—H3B	0.8600	C1—C2	1.478 (4)
N1—C9	1.439 (3)	C2—C3	1.368 (4)
N1—C8	1.384 (4)	C2—C7	1.378 (5)
N1—C1	1.396 (4)	C3—C4	1.382 (5)
C11—C17	1.463 (4)	C4—C5	1.367 (7)
C11—C12	1.380 (4)	C5—C6	1.376 (5)
C11—C16	1.380 (4)	C6—C7	1.368 (5)
C12—C13	1.397 (4)	C7—C8	1.481 (4)
C13—C14	1.379 (5)	C9—C10	1.507 (4)
C14—C15	1.372 (6)	C3—H3	0.9300
C15—C16	1.366 (5)	C4—H4	0.9300
C18—C19	1.495 (4)	C5—H5	0.9300
C19—C23	1.383 (3)	C6—H6	0.9300
C19—C20	1.374 (3)	C9—H9A	0.9700
C20—C21	1.378 (4)	C9—H9B	0.9700
			
C12—O5—C24	118.3 (2)	N4—C21—H21	118.00
C10—O3—H3A	110.00	N4—C22—H22	118.00
N3—N2—C17	115.7 (2)	C23—C22—H22	118.00
N2—N3—C18	118.0 (2)	C22—C23—H23	121.00
C21—N4—C22	116.9 (2)	C19—C23—H23	121.00
C18—N3—H3B	121.00	O5—C24—H24C	109.00
N2—N3—H3B	121.00	H24B—C24—H24C	110.00
C1—N1—C9	123.6 (3)	H24A—C24—H24B	110.00
C1—N1—C8	111.8 (2)	H24A—C24—H24C	109.00
C8—N1—C9	124.3 (3)	O5—C24—H24B	109.00
C12—C11—C16	118.7 (3)	O5—C24—H24A	110.00
C16—C11—C17	120.9 (2)	O1—C1—N1	124.2 (3)
C12—C11—C17	120.4 (3)	O1—C1—C2	130.2 (3)
C11—C12—C13	120.6 (3)	N1—C1—C2	105.6 (3)
O5—C12—C13	123.3 (2)	C1—C2—C3	129.8 (3)
O5—C12—C11	116.1 (2)	C1—C2—C7	108.6 (3)
C12—C13—C14	118.4 (3)	C3—C2—C7	121.6 (3)
C13—C14—C15	121.7 (3)	C2—C3—C4	117.5 (3)
C14—C15—C16	118.8 (3)	C3—C4—C5	120.9 (3)
C11—C16—C15	121.8 (3)	C4—C5—C6	121.6 (4)
N2—C17—C11	119.4 (3)	C5—C6—C7	117.6 (4)
N3—C18—C19	116.0 (2)	C2—C7—C8	107.8 (3)
O6—C18—C19	120.7 (2)	C6—C7—C8	131.3 (3)
O6—C18—N3	123.2 (2)	C2—C7—C6	120.9 (3)
C18—C19—C23	122.3 (2)	O2—C8—C7	129.5 (3)
C20—C19—C23	118.4 (2)	N1—C8—C7	106.3 (3)
C18—C19—C20	119.2 (2)	O2—C8—N1	124.2 (3)
C19—C20—C21	118.6 (2)	N1—C9—C10	112.1 (2)
N4—C21—C20	123.7 (2)	O3—C10—C9	111.3 (2)
N4—C22—C23	123.6 (3)	O4—C10—C9	123.6 (2)
C19—C23—C22	118.8 (2)	O3—C10—O4	125.1 (3)
C12—C13—H13	121.00	C2—C3—H3	121.00
C14—C13—H13	121.00	C4—C3—H3	121.00
C15—C14—H14	119.00	C3—C4—H4	120.00
C13—C14—H14	119.00	C5—C4—H4	120.00
C16—C15—H15	121.00	C4—C5—H5	119.00
C14—C15—H15	121.00	C6—C5—H5	119.00
C11—C16—H16	119.00	C5—C6—H6	121.00
C15—C16—H16	119.00	C7—C6—H6	121.00
N2—C17—H17	120.00	N1—C9—H9A	109.00
C11—C17—H17	120.00	N1—C9—H9B	109.00
C21—C20—H20	121.00	C10—C9—H9A	109.00
C19—C20—H20	121.00	C10—C9—H9B	109.00
C20—C21—H21	118.00	H9A—C9—H9B	108.00
			
C24—O5—C12—C11	175.1 (3)	O6—C18—C19—C23	142.3 (3)
C24—O5—C12—C13	−3.7 (5)	O6—C18—C19—C20	−32.7 (4)
C17—N2—N3—C18	−167.0 (3)	N3—C18—C19—C23	−34.9 (4)
N3—N2—C17—C11	−175.9 (2)	N3—C18—C19—C20	150.1 (3)
N2—N3—C18—O6	1.1 (4)	C18—C19—C23—C22	−173.1 (3)
N2—N3—C18—C19	178.2 (2)	C23—C19—C20—C21	−0.3 (4)
C22—N4—C21—C20	1.6 (4)	C18—C19—C20—C21	174.9 (3)
C21—N4—C22—C23	0.2 (4)	C20—C19—C23—C22	1.9 (4)
C1—N1—C9—C10	−87.7 (4)	C19—C20—C21—N4	−1.6 (5)
C8—N1—C9—C10	85.4 (3)	N4—C22—C23—C19	−1.9 (4)
C8—N1—C1—C2	0.9 (3)	O1—C1—C2—C3	0.1 (6)
C9—N1—C1—O1	−4.7 (5)	O1—C1—C2—C7	178.7 (3)
C9—N1—C1—C2	174.7 (3)	N1—C1—C2—C3	−179.2 (3)
C1—N1—C8—O2	179.4 (3)	N1—C1—C2—C7	−0.7 (3)
C1—N1—C8—C7	−0.8 (3)	C1—C2—C3—C4	177.8 (3)
C9—N1—C8—O2	5.7 (5)	C7—C2—C3—C4	−0.6 (5)
C8—N1—C1—O1	−178.5 (3)	C1—C2—C7—C6	−178.6 (3)
C9—N1—C8—C7	−174.5 (2)	C1—C2—C7—C8	0.2 (3)
C17—C11—C12—C13	−175.8 (3)	C3—C2—C7—C6	0.1 (5)
C12—C11—C16—C15	−2.3 (5)	C3—C2—C7—C8	178.9 (3)
C17—C11—C12—O5	5.4 (4)	C2—C3—C4—C5	1.1 (5)
C12—C11—C17—N2	−165.6 (3)	C3—C4—C5—C6	−1.1 (6)
C16—C11—C17—N2	16.3 (4)	C4—C5—C6—C7	0.6 (5)
C17—C11—C16—C15	175.9 (3)	C5—C6—C7—C2	−0.1 (5)
C16—C11—C12—O5	−176.4 (3)	C5—C6—C7—C8	−178.6 (3)
C16—C11—C12—C13	2.4 (5)	C2—C7—C8—O2	−179.9 (3)
C11—C12—C13—C14	−1.1 (5)	C2—C7—C8—N1	0.3 (3)
O5—C12—C13—C14	177.7 (3)	C6—C7—C8—O2	−1.3 (6)
C12—C13—C14—C15	−0.5 (6)	C6—C7—C8—N1	179.0 (3)
C13—C14—C15—C16	0.7 (6)	N1—C9—C10—O3	176.9 (3)
C14—C15—C16—C11	0.8 (5)	N1—C9—C10—O4	−3.0 (5)

### Hydrogen-bond geometry (Å, º)

**Table:**

*D*—H···*A*	*D*—H	H···*A*	*D*···*A*	*D*—H···*A*
O3—H3*A*···N4^i^	0.82	1.86	2.681 (3)	177
N3—H3*B*···O6^ii^	0.86	2.12	2.958 (3)	164
C4—H4···O4^iii^	0.93	2.44	3.190 (4)	138
C20—H20···O3^ii^	0.93	2.57	3.500 (4)	174

Symmetry codes: (i) *x*−1/2, −*y*+1/2, *z*−3/2; (ii) *x*+1/2, −*y*+1/2, *z*+1/2; (iii) −*x*+1, −*y*, −*z*.

## References

[bb1] Bijev, A. (2006). *Lett. Drug. Des. Discov.* **3**, 506–512.

[bb2] Bruker (2005). *SADABS* Bruker AXS Inc., Madison, Wisconsin, USA.

[bb3] Bruker (2009). *APEX2* and *SAINT* Bruker AXS Inc., Madison, Wisconsin, USA.

[bb4] Farrugia, L. J. (1997). *J. Appl. Cryst.* **30**, 565.

[bb5] Farrugia, L. J. (1999). *J. Appl. Cryst.* **32**, 837–838.

[bb6] Imramovský, A., Polanc, S., Vinšová, J., Kočevar, M., Jampílek, J., Rečková, Z. & Kaustová, J. (2007). *Bioorg. Med. Chem.* **15**, 2551–2559. 10.1016/j.bmc.2007.01.051 17306980

[bb7] Maccari, R., Ottana, R. & Vigorita, M. G. (2005). *Bioorg. Med. Chem. Lett.* **15**, 2509–2513. 10.1016/j.bmcl.2005.03.065 15863306

[bb8] McNamara, D. P., Childs, S. L., Giordano, J., Iarriccio, A., Cassidy, J., Shet, M. S., Mannion, R., O’Donnell, E. & Park, A. (2006). *Pharm. Res.* **23**, 1888–1897. 10.1007/s11095-006-9032-3 16832611

[bb9] Peterson, M. L., Hickey, M. B., Zaworotko, M. J. & Almarsson, O. (2006). *J. Pharm. Pharm. Sci.* **9**, 317–326. 17207415

[bb10] Schultheiss, N. & Newman, A. (2009). *Cryst. Growth Des.* **9**, 2950–2967. 10.1021/cg900129f PMC2690398 19503732

[bb11] Sheldrick, G. M. (2008). *Acta Cryst.* A**64**, 112–122. 10.1107/S0108767307043930 18156677

[bb12] Shindikar, A. V. & Viswanathan, C. L. (2005). *Bioorg. Med. Chem. Lett.* **15**, 1803–1806. 10.1016/j.bmcl.2005.02.037 15780610

[bb13] Sinha, N., Jain, S., Tilekar, A., Upadhayaya, R. S., Kishore, N., Jana, G. H. & Arora, S. K. (2005). *Bioorg. Med. Chem. Lett.* **15**, 1573–1576. 10.1016/j.bmcl.2005.01.073 15745799

[bb14] Spek, A. L. (2009). *Acta Cryst.* D**65**, 148–155. 10.1107/S090744490804362X PMC2631630 19171970

[bb15] Sriram, D., Yogeeswari, P. & Madhu, K. (2006). *Bioorg. Med. Chem.* **14**, 876–878. 10.1016/j.bmcl.2005.11.004 16303302

[bb16] Vishweshwar, P., McMahon, J. A. & Bis, J. A. (2009). *J. Pharm. Sci.* **95**, 499–516. 10.1002/jps.20578 16444755

